# Ghrelin enhances tubular magnesium absorption in the kidney

**DOI:** 10.3389/fphys.2024.1363708

**Published:** 2024-04-04

**Authors:** Mingzhu Nie, Jing Zhang, Manjot Bal, Claudia Duran, Sung Wan An, Jeffrey M. Zigman, Michel Baum, Chitkale Hiremath, Denise K. Marciano, Matthias T. F. Wolf

**Affiliations:** ^1^ Pediatric Nephrology, Department of Pediatrics, University of Texas Southwestern Medical Center, Dallas, TX, United States; ^2^ Pediatric Nephrology, Department of Pediatrics, University of Michigan, Ann Arbor, MI, United States; ^3^ Department of Internal Medicine, Center for Hypothalamic Research, UTSW Medical Center, Dallas, TX, United States; ^4^ Department of Internal Medicine, Nephrology, and Department of Cell Biology, UTSW Medical Center, Dallas, TX, United States

**Keywords:** magnesium, TRPM6/7, Ghrelin, GHSR, protein kinase A

## Abstract

Osteoporosis after bariatric surgery is an increasing health concern as the rate of bariatric surgery has risen. In animal studies mimicking bariatric procedures, bone disease, together with decreased serum levels of Ca^2+^, Mg^2+^ and the gastric hormone Ghrelin were described. Ghrelin regulates metabolism by binding to and activating the growth hormone secretagogue receptor (GHSR) which is also expressed in the kidney. As calcium and magnesium are key components of bone, we tested the hypothesis that Ghrelin-deficiency contributes to osteoporosis via reduced upregulation of the renal calcium channel TRPV5 and the heteromeric magnesium channel TRPM6/7. We expressed GHSR with TRPV5 or TRPM6/7 channel in HEK293 cells and treated them with purified Ghrelin. Whole-cell current density was analyzed by patch-clamp recording. Nephron-specific gene expression was performed by tubular microdissection followed by qPCR in wild-type (WT) mice, and immunofluorescent imaging of GHSR-eGFP mice. Tubular magnesium homeostasis was analyzed in GHSR-null and WT mice at baseline and after caloric restriction. After Ghrelin exposure, whole-cell current density did not change for TRPV5 but increased for TRPM6/7 in a dose-dependent fashion. Applying the Ghrelin-mimetic (D-Trp^7^, Ala^8^,D-Phe^10^)-α-MSH (6–11) amide without and with the GHSR antagonist (D-Lys^3^)-GHRP6, we confirmed the stimulatory role of Ghrelin towards TRPM6/7. As GHSR initiates downstream signaling via protein kinase A (PKA), we found that the PKA inhibitor H89 abrogated TRPM6/7 stimulation by Ghrelin. Similarly, transfected Gα_s_, but not the Gα_s_ mutant Q227L, nor Gα_i2_, Gα_q_, or Gα_13_ upregulated TRPM6/7 current density. In microdissected TALs and DCTs similar levels of GHSR mRNA were detected. In contrast, TRPM6 mRNA was expressed in the DCT and also detected in the TAL at 25% expression compared to DCT. Immunofluorescent studies using reporter GHSR-eGFP mice showed a strong eGFP signal in the TAL but surprisingly displayed no eGFP signal in the DCT. In 3-, 6-, and 9-month-old GHSR-null and WT mice, baseline serum magnesium was not significantly different, but 24-h urinary magnesium excretion was elevated in 9-month-old GHSR-null mice. In calorically restricted GHSR-null mice, we detected excess urinary magnesium excretion and reduced serum magnesium levels compared to WT mice. The kidneys from calorically restricted WT mice showed upregulated gene expression of magnesiotropic genes *Hnf1b*, *Cldn-16*, *Cldn-19*, *Fxyd-2b*, and *Parvalbumin* compared to GHSR-null mice. Our *in vitro* studies show that Ghrelin stimulates TRPM6/7 via GHSR and Gα_s_-PKA signaling. The murine studies are consistent with Ghrelin-GHSR signaling inducing reduced urinary magnesium excretion, particularly in calorically restricted mice when Ghrelin levels are elevated. This effect may be mediated by Ghrelin-upregulation of TRPM6 in the TAL and/or upregulation of other magnesiotropic genes. We postulate that rising Ghrelin levels with hunger contribute to increased renal Mg^2+^ reabsorption to compensate for lack of enteral Mg^2+^ uptake.

## 1 Introduction

With more than 400 million obese adults worldwide, obesity has become an epidemic condition in the United States and around the world. Obesity contributes significantly to other co-morbidities such as type 2 diabetes mellitus (T2DM) and hypertension and consumes a significant proportion of American healthcare spending (Haslam and James, 2005; Ogden et al., 2006). Bariatric surgery has emerged as an effective treatment for obesity-related conditions, and increased almost six-fold between 1998 and 2002 (Schauer et al., 2014). These procedures have short-term beneficial effects regarding control of T2DM, hyperlipidemia, and hyperuricemia (Schauer et al., 2012; Schauer et al., 2014). Long-term complications of these procedures are far less well understood but include osteopenia and increased fracture risk (von Mach et al., 2004; Berarducci et al., 2009). So far, bariatric surgery-related osteopenia is thought to be mainly caused by mechanical and nutritional effects but is incompletely understood (Folli et al., 2012).

Weight loss after bariatric surgery is caused by many different mechanisms. The orexigenic hormone Ghrelin is a strong mediator of appetite. Serum Ghrelin is reduced after several forms of bariatric surgery thus likely contributing to decreased food intake from a potent reduction in appetite (Malkani, 2015). Ghrelin is a 28-amino acid peptide secreted into circulation from cells present within the lining of the gastroenteral tract, predominantly the gastric fundus (Kojima et al., 1999). Levels of circulating Ghrelin, which potently stimulates food intake, are elevated pre-prandially and reduced postprandially (Cummings et al., 2001). Postsurgical Ghrelin production decreases when the gastric fundus is manipulated after certain bariatric procedures, such as Roux-en-Y-gastric bypass, sleeve gastrectomy, and biliopancreatic diversion (Geloneze et al., 2003; Kotidis et al., 2006; Thomas and Schauer, 2010). Ghrelin is acylated and binds to the growth hormone secretagogue receptor (GHSR), a G protein-coupled receptor which is expressed in multiple organs, most notably the brain, the pituitary gland, and the pancreatic islets (Zigman et al., 2006; Gupta et al., 2021). GHSR expression is also found in the kidney (Hosoda et al., 2000; Dagli et al., 2009). Within the kidney, GHSR localizes to distal nephrons and stimulates the epithelial Na^+^ channel ENaC (Venables et al., 2011; Kemp et al., 2013).

Interestingly, animals undergoing gastric fundectomy also develop osteopenia, alike patients after certain forms of bariatric surgery. In rats, gastric fundectomy leads to osteopenia and a significantly lower bone content of calcium (Ca^2+^) and magnesium (Mg^2+^) (Rumenapf et al., 1998). In pigs, gastric fundectomy also induces osteopenia, a lower Ca^2+^ bone content, lower Ghrelin levels, and a significant decrease in serum Ca^2+^ and Mg^2+^ levels (Tatara et al., 2007).

The importance of Ca^2+^ in bone formation and bone health is well known. The kidney plays a crucial role in Ca^2+^ homeostasis by reabsorbing 95%–98% of filtered Ca^2+^(Costanzo and Windhager, 1978; Frick and Bushinsky, 2003). The vast majority of filtered Ca^2+^ is reabsorbed in the proximal tubule (PT) (65%) and in the TAL (25%) via the paracellular pathway coupled to Na^+^ absorption. The remaining Ca^2+^ reabsorption occurs in the late distal convoluted tubule (DCT) and connecting tubule (CNT) via transcellular transport, which includes Ca^2+^ entry through the transient receptor potential vanilloid channel TRPV5, which is localized in the apical membrane and interferes with bone health in mice if deficient (Hoenderop et al., 2003).

The role of Mg^2+^ in bone health is less well understood. Mg^2+^ is the second most abundant intracellular cation (de Baaij et al., 2015). The vast majority of total body Mg^2+^ is stored in bone (Alfrey et al., 1974; de Baaij et al., 2015). Mg^2+^ is involved with bone metabolism by inducing osteoblast proliferation (Xie et al., 2023). Mg^2+^-deficient animals have impaired bone growth, decreased bone strength, decreased osteoblasts, and increased osteoclast numbers (Boskey et al., 1992; Rude et al., 2003; Rude and Gruber, 2004). Approximately 80% of the total plasma Mg^2+^ is filtered by the glomerulus and about 95%–99% of the filtered Mg^2+^ is reabsorbed (de Baaij et al., 2015). Even though the DCT reabsorbs only 5%–10% of Mg^2+^, this is the nephron segment which determines the final urinary Mg^2+^ concentration in an active, transcellular, and regulated fashion via the apical heterodimeric epithelial magnesium channel complex consisting of transient receptor potential melastatin type 6 (TRPM6) and 7 (TRPM7) (Hoenderop and Bindels, 2008). Deletion of *Trpm6* in mice is embryonic lethal, whereas humans with recessive mutations in *TRPM6* develop early onset of hypomagnesemia and secondary hypocalcemia, further emphasizing the significance of Mg^2+^ reabsorption in the DCT (Schlingmann et al., 2002; Walder et al., 2009).

Given that osteopenia develops after bariatric surgery in animal models and humans together with lower serum Ghrelin levels, we hypothesized that the reduction in serum Ghrelin secretion post-surgery may impair tubular Ca^2+^ or Mg^2+^ absorption via TRPV5 or TRPM6/7 which subsequently leads to reduced Ca^2+^ and Mg^2+^ bone content and osteopenia. Therefore, we tested if the gastric hormone Ghrelin enhances renal Ca^2+^ or Mg^2+^ homeostasis via the Ca^2+^ channel TRPV5 or the heterodimeric Mg^2+^ channel TRPM6/7 *in vitro* applying whole-cell patch clamp recording and *in vivo* using wild-type (WT) and GHSR-null mice.

## 2 Materials and methods

### 2.1 Materials and DNA constructs

GFP-tagged rabbit TRPV5 cDNA cloned into pEGFP3-N3 vector was used as previously published (Nie et al., 2016). Human TRPM6 was cloned into the bicistronic vector pCINeo/IRES-GFP (Nair et al., 2012). TRPM7 is ubiquitously expressed in HEK293 cells and interacts with TRPM6, therefore we will refer to this heterodimeric channel as TRPM6/7. Human TRPM6 and TRPM7 plasmids were kindly provided by Drs. Chubanov and Gudermann, Ludwig-Maximilians-University Munich, Germany. Human GHSR cDNA cloned into pcDNA3.1 was obtained from the University of Missouri-Rolla cDNA Resource Center (Rolla, MO). Plasmids for Gα_s_, Gα_i2_, Gα_q_, Gα_12/13_, and the Gα_s_ mutant Q227L were kindly provided by Richard T. Miller, UT Southwestern Medical School Dallas. Purified human Ghrelin (#031–30, dissolved in water) was purchased from Phoenix Pharmaceuticals (Burlingame, CA). We applied a dose of 100 nM and evaluated the effect after 1 h if not described differently in the text. The Ghrelin-mimetic (D-Trp^7^,Ala^8^,D-Phe^10^)-α-MSH (6–11) amide (#4008401, dissolved in water) and the GHSR antagonist (D-Lys^3^)-GHRP6 (#H-3108, dissolved in water) were both obtained from Bachem (Bubendorf, Switzerland). We bought the PKA inhibitor H89 dihydrochloride hydrate (#B1427, dissolved in DMSO) from Sigma-Aldrich (St. Louis, MO). Antibody against TRPM6 (ACC-046) (host rabbit, 1:1000) was purchased from Alomone (Jerusalem, Israel). Antibody against Claudin-16 (host rabbit, 1:500) was kindly provided by J. Hou (St. Louis). Antibodies against β-actin (C4) (sc-47778) (1:500), IgG kappa binding protein conjugated to Horseradish Peroxidase (m-IgGκ BP-HRP) (sc-516102), and goat anti-rabbit-IgG-HRP (sc-2025) (1:2000), were purchased from Santa Cruz Biotechnology (Dallas, TX).

### 2.2 Cell culture and transfection

HEK293 cells were cultured as previously published (Nie et al., 2018). The cell lines present in this study were obtained from American Type Culture Collection (Manassas, VA). Cells were transiently transfected using Lipofectamine 2000^®^ reagent (Thermo Fisher Scientific, Waltham, MA) with plasmids (2 μg per 6-well) containing TRPV5-EGFP, or GFP-TRPM6, together with GHSR, Gα_s_, Gα_i2_, Gα_q_, Gα_13_, the Gα_s_ mutant Q227L, or control vectors as indicated, in each experiment. In each experiment the total amount of DNA for transfection was balanced by using empty vectors.

### 2.3 Quantitative RT-PCR studies

Total RNA was isolated from kidneys from WT and GHSR-null mice using miRNeasy Mini kits from Qiagen (Germantown, MD). First-strand cDNA was synthesized by iScript™ cDNA synthesis kit (Bio-Rad, Hercules, CA). Relative transcript expression was measured by quantitative real-time PCR using iTaq™ Universal SYBR^®^ Green Supermix (Bio-Rad, Hercules, CA). Samples were run on CFX96 Real-Time PCR Detection System (Bio-Rad, Hercules, CA). 18S RNA was used to normalize for expression of mRNA. Primers for qRT-PCR were previously published (van Angelen et al., 2013). Data were analyzed using the Bio-Rad CXF software.

### 2.4 Immunoblotting

The kidney lysates were collected using a buffer containing 25 mM Tris-HCl pH7.6, 150 mM NaCl, 1% NP-40, 0.1% SDS with protease inhibitor (Thermo Fisher Scientific, Waltham, MA). The protein concentration was measured with the Pierce Bicinchoninic acid reagent (Thermo Fisher Scientific, Waltham, MA). An equal amount of protein was loaded for SDS-PAGE and transferred onto PVDF membrane. The membranes were then incubated sequentially with a blocking solution containing 5% nonfat milk, primary antibody incubation and finally horseradish peroxidase-conjugated secondary antibody. The antigens on the blots were revealed using the enhanced chemiluminescence (ECL) kit (Bio-Rad, Hercules, CA) to record signals by ChemiDoc MP imaging system (Bio-Rad, Hercules, CA). The densitometry of the blots was performed with Image Lab Software Ver 6.1.0 (Bio-Rad, Hercules, CA).

### 2.5 Whole-cell patch-clamp recording

For individual TRPM6, TRPM7, and combined TRPM6/7 current density plasmids from Drs. Chubanov and Gudermann from the Walther-Straub Institute of Pharmacology and Toxicology, Ludwig-Maximilians-University Munich, were used. To examine whole-cell patch clamp recording for TRPM6/7 we transfected cells with TRPM6-IRES-GFP using endogenous TRPM7 expression to create the heteromeric dimer TRPM6/7 as was previously outlined (Nie et al., 2016; Nie et al., 2018). Approximately 48 h after transfection cells were dissociated and placed in a chamber for ruptured whole-cell recordings as described previously. Transfected cells were identified for recording by their GFP fluorescence. Pipette and bath solutions for TRPV5 were used as described earlier (Nie et al., 2016). TRPM6/7 bath solution contained (in mM) 140 NaCl, 5 CsCl, 2 CaCl_2_, 1 MgCl_2_, 10 glucose, 10 HEPES (pH 7.4 with NaOH). The pipette TRPM6/7 solution contained (in mM) 120 CsCl, 10 NaCl, 1 HEDTA, and 10 HEPES, (pH 7.2 with CsOH). Whole-cell patch clamp pipettes were pulled from borosilicate glass (Dagan Corporation, Minneapolis, MN) and had resistance between 1.5 and 3 MΩ. The cell membrane capacitance and series resistance were monitored and compensated (>75%) electronically using an Axopatch 200B amplifier (Axon Instruments, Foster City, CA). Voltage protocol consists of 0 mV holding potential and successive voltage sets (400-ms duration) from −100 to +100 mV in +20 increments. Current densities were obtained by normalizing current amplitude (obtained at +100 mV for TRPM6/7) to cell capacitance. Data acquisition was performed using ClampX9.2 software (Axon Instruments). Currents were low-pass filtered at 2 kHz using eight-pole Bessels filter in the clamp amplifier, sampled every 0.1 ms (10 kHz) with Digidata-1440 interface, and stored directly to a computer hard drive.

### 2.6 Immunofluorescent staining

GHSR-eGFP transgenic mice were obtained from the Mouse Mutant Regional Resource Center (MMRC) Repository at the University of California Davis [Tg (Ghsr-EGFP)KZ65G-sat; RRID:IMSR_MMRRC:030,942]. The mouse line was generated on an FVB/N-Crl:CD1(ICR) genetic background. The mouse was previously back-crossed to the C57BL/6 background (Smith et al., 2013). Anesthetized mice were perfused with 4% w/v paraformaldehyde in PBS (pH7.4). Kidneys were harvested and sectioned. Sections were blocked with 10% v/v donkey sera in PBS and immunofluorescence performed with primary antibodies overnight at 4°C (Groenestege et al., 2006). The following primary antibodies were utilized: GFP (GFP-1020, Aves Labs, 1:500), AQP2 (SBT, sc-9882, 1:100), LTL (Vector Laboratories, B-1325, 1:500), THP (Thermo-Fischer, PA5-47706, 1:200), and NCC (Millipore, AB3553, 1:100). Fluorescent images were obtained using a Zeiss LSM510 confocal microscope (Zeiss, Jena, Germany). All animal experiments were performed in compliance with relevant laws and institutional guidelines and were approved by the UT Southwestern Medical Center at Dallas Institutional Animal Care and Use Committee (IACUC protocol #2017–102144).

### 2.7 Metabolic cage studies in mice

Urinary excretion of Mg^2+^ and serum Mg^2+^ was determined in 3-, 6-, and 9-month-old male WT and GHSR-null mice on a C57BL/6 background. Animals were acclimated for 3 days in metabolic cages prior to experiments. Urine was collected for 4 days, and the last collection was used for analysis. Blood was obtained on the fourth day of collection. Daily urine volume was measured after bladder massage (Hoorn et al., 2011). Serum Mg^2+^ was tested by the UT Southwestern Metabolic Phenotyping core. Urine Mg^2+^ was analyzed by the UT Southwestern O’Brien Kidney Research Center in Dallas.

### 2.8 Studies in calorically restricted mice

We followed a calory restriction similar to a previously published protocol (Zhang et al., 2015). Specifically, we first assessed an average caloric food intake per animal over 5 days in metabolic cages. Subsequently, we provided over the next 8 days a 60% caloric restriction by providing 40% of the average caloric intake from the previous 5 days. Animals were provided with food for only 1 hour at ∼6 PM. Animals were monitored applying the body condition score (Ullman-Cullere and Foltz, 1999), muscle mass and fat, and weight. Animals were excluded from the study if they had a body condition score below 1. There was no fluid restriction. After caloric restriction for 8 days animals were fasted for 24 h. Afterwards, we collected stool and urine samples to measure daily urinary and fecal Mg^2+^ excretion.

### 2.9 Statistical analysis

Student's t-tests were used to test if there are significant differences in the continuous outcomes between two study groups. For multiple comparisons one-way ANOVA studies followed by Student-Newman-Keuls method, allowing for pairwise multiple comparisons, were performed. Data are reported as means ± S.D. *p* < 0.05 was considered statistically significant.

## 3 Results

### 3.1 Ghrelin stimulates the Mg^2+^ channel TRPM6/7 but not the Ca^2+^ channel TRPV5

**FIGURE 1:**
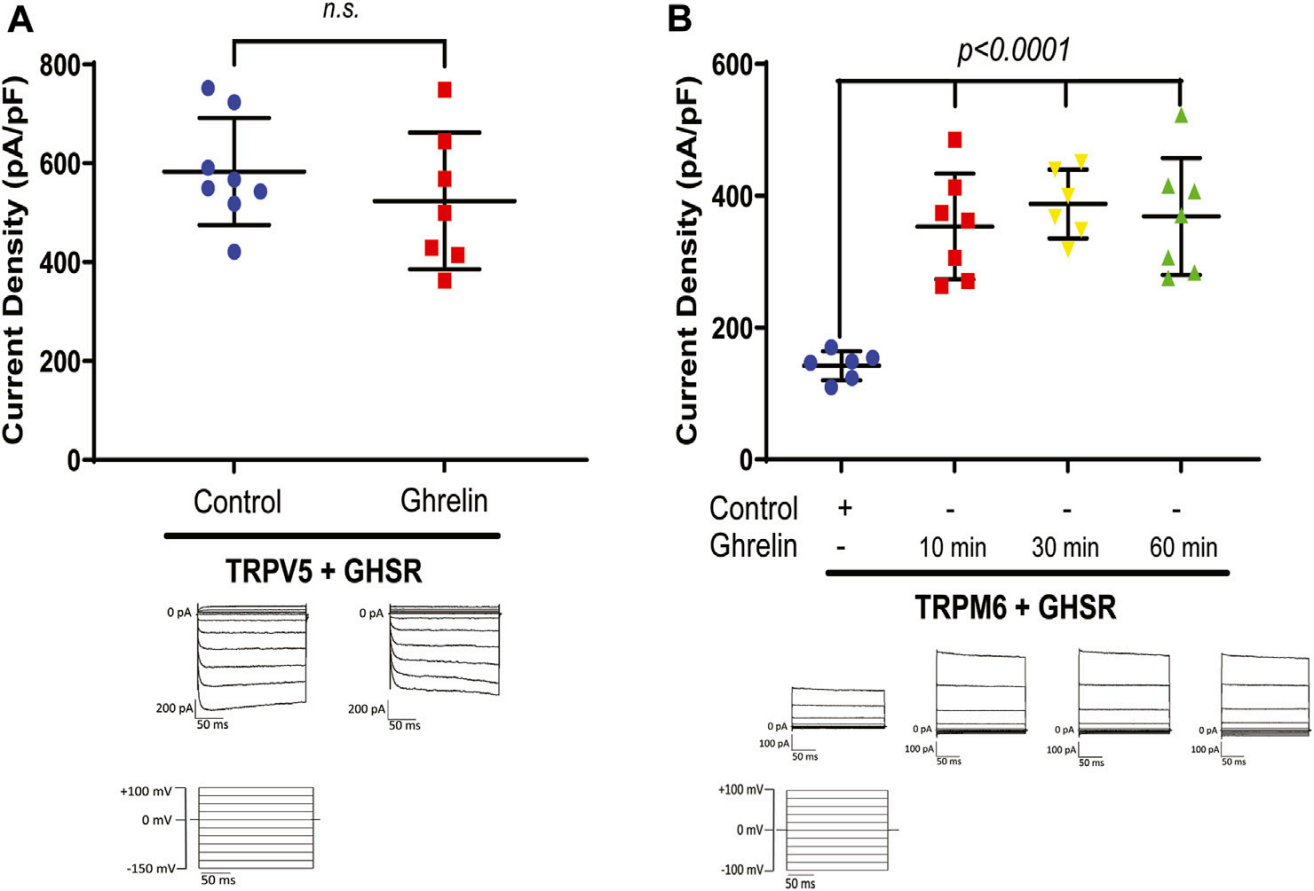
**(A)** The Ca^2+^ channel TRPV5 is not stimulated by purified Ghrelin when co-expressed with GHSR in HEK293 cells (TRPV5+GHSR + control 583.2 ± 108.2 pA/pF vs. TRPV5+GHSR + Ghrelin vs. 524 ± 138 pA/pF; *n. s.*, n = six to eight each group). Typical current traces are shown below each specific group. Applied test pulses are shown at the bottom. **(B)** In contrast, the Mg^2+^ channel TRPM6/7 displays increased current density when co-expressed with GHSR and treated with purified Ghrelin after 10, 30, and 60 min (TRPM6+GHSR + control 142.3 ± 21.9 pA/pF vs. TRPM6+GHSR + Ghrelin (10 min) 353.4 ± 80.2 pA/pF, *p < 0.0001*, vs. TRPM6+GHSR + Ghrelin (30 min) 387.6 ± 52.3 pA/pF, *p < 0.0001*, TRPM6+GHSR + Ghrelin (60 min) 368.6 ± 88.6 pA/pF, *p < 0.0001*, n = six to seven each group). Typical current traces are shown below each specific group. Applied test pulses are shown at the bottom.

Given the known association between bariatric surgery and bone disease, and the known significance of Ca^2+^ for bone integrity, we first tested if Ghrelin has any stimulatory effect on the Ca^2+^ channel TRPV5 via GHSR. Applying whole-cell patch clamp recording on HEK293 cells treated with purified Ghrelin and transfected with TRPV5-GFP and GHSR we did not identify any significant increase of TRPV5 current density by Ghrelin (Figure 1A). However, when we studied the effect of purified Ghrelin on HEK293 cells co-transfected with TRPM6-GFP and GHSR we noticed an almost two-fold current density stimulation (*p < 0.0001*) as early as 10 min which was sustained at 30 and 60 min (Figure 1B). This finding was consistent with a stimulatory role of Ghrelin towards the Mg^2+^ channel TRPM6/7 via GHSR *in vitro*. In our experience, baseline transfected TRPM6 current density is very low with higher current density for transfected TRPM7, while combined TRPM6/7 has the highest current density (Supplementary Figure S1). Because TRPM7 is ubiquitously expressed, and we overexpressed TRPM6 it is very likely that we analyzed the current density for the hetermeric dimer TRPM6/7 which reflects the physiological situation in the gastrointestinal tract and the kidney.

### 3.2 A Ghrelin-mimetic confirms TRPM6/7 stimulation which is inhibited by a GHSR blocker

**FIGURE 2:**
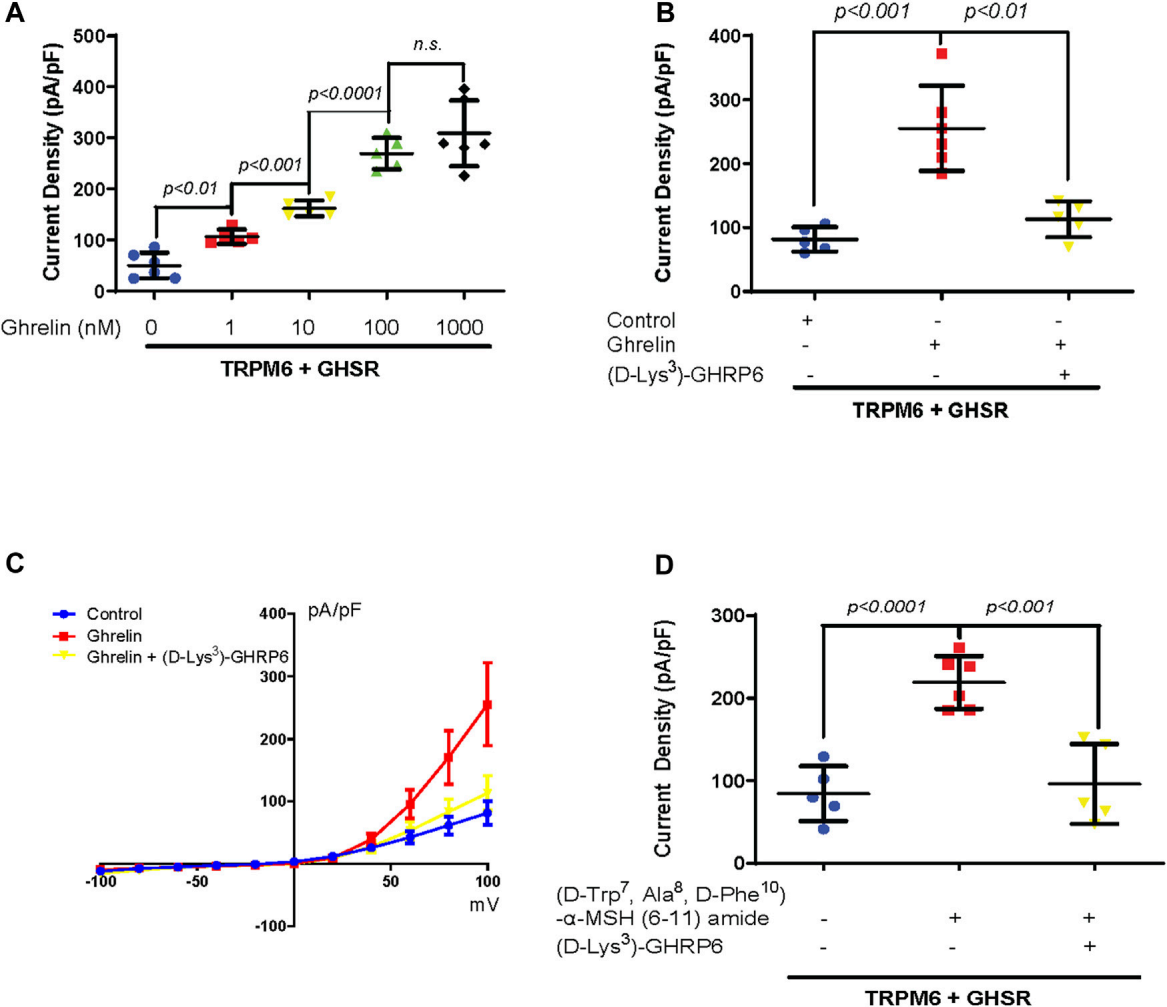
**(A)** A dose-dependent increase in TRPM6/7 current density occurs with purified Ghrelin treatment at dosages from 1 to 100 nM 60 min after treatment (TRPM6+GHSR+0 nM Ghrelin 49.8 ± 25.2 pA/pF vs. TRPM6+GHSR+1 nM Ghrelin 106.4 ± 14.1 pA/pF, *p < 0.01*, vs. TRPM6+GHSR+10 nM Ghrelin 161.9 ± 15.4 pA/pF, *p < 0.001*, vs. TRPM6+GHSR+100 nM Ghrelin 269.4 ± 30.6 pA/pF, *p < 0.0001*, n = five to six each group). No significant rise in TRPM6/7 current density is observed when the dose increased from 100 to 1000 nM (TRPM6+GHSR+100 nM Ghrelin 269.4 ± 30.6 pA/pF vs. TRPM6+GHSR+1000 nM Ghrelin 309.2 ± 64.3 pA/pF, *n. s*, n = five to six each group). **(B)** The stimulation of TRPM6/7 by Ghrelin is blocked by the GHSR-antagonist (D-Lys^3^)-GHRP6 (TRPM6+GHSR + control 81.5 ± 19.2 pA/pF vs. TRPM6+GHSR + Ghrelin 255.3 ± 66.4 pA/pF, *p < 0.001*, vs. TRPM6+GHSR + Ghrelin+(D-Lys^3^)-GHRP6 113.2 ± 28 pA/pF, *p < 0.01*, n = five to six each group). **(C)** TRPM6/7 current density is evoked by test pulses from −100 to +100 mV with 20 mV increments. The current–voltage (I/V) curves display a characteristic outwardly rectifying current typical for TRPM6. The I/V curve demonstrated more activity with Ghrelin stimulation as analyzed in **(B)**. **(D)**. TRPM6/7 current density is stimulated by the Ghrelin-mimetic (D-Trp^7^, Ala^8^,D-Phe^10^)-α-MSH (6–11) amide (TRPM6+GHSR + control 84.3 ± 33.3 pA/pF vs. TRPM6+GHSR+(D-Trp^7^, Ala^8^,D-Phe^10^)-α-MSH (6–11) amide 218.9 ± 31.9 pA/pF, *p < 0.0001*). This activation of TRPM6/7 current density is blocked by (D-Lys^3^)-GHRP6 (TRPM6+GHSR+(D-Trp^7^, Ala^8^,D-Phe^10^)-α-MSH (6–11) amide 218.9 ± 31.9 pA/pF vs. TRPM6+GHSR+(D-Trp^7^, Ala^8^,D-Phe^10^)-α-MSH (6–11) amide +(D-Lys^3^)-GHRP6 96.1 ± 48.5 pA/pF, *p < 0.001*, n = five to six each group).

As a next step we performed a dose response curve for the effect of Ghrelin on TRPM6/7 (Figure 2A). For Ghrelin dosages ranging from 1 to 100 nM we found significant increases in TRPM6/7 whole-cell current density while for the dose increase from 100 nM to 1000 nM no further significant rise in current density was noticed suggesting that a saturation of the Ghrelin effect occurred (Figure 2A). To confirm the stimulatory role of Ghrelin via GHSR on TRPM6/7 we studied if this effect could be blocked by the GHSR inhibitor (D-Lys^3^)-GHRP6 (Figure 2B). While Ghrelin enhanced TRPM6/7 whole-cell current density as anticipated (*p < 0.001*), adding (D-Lys^3^)-GHRP6 significantly decreased the Ghrelin-mediated stimulation of TRPM6/7 (*p < 0.01*) (Figure 2B). Our current–voltage (I/V) curves for this experiment confirmed the characteristic outwardly rectifying current for TRPM6/7+Ghrelin which was significantly lower for the control and (D-Lys^3^)-GHRP6+Ghrelin groups (Figure 2C). Finally, we examined if the Ghrelin-mimetic (D-Trp^7^, Ala^8^, D-Phe^10^)-α-MSH (6–11) amide can also stimulate TRPM6/7. We identified an approximately 2.5-fold stimulatory effect for the Ghrelin-mimetic towards TRPM6/7 (*p < 0.0001*) which was then blocked by the GHSR inhibitor (D-Lys^3^)-GHRP6 (*p < 0.001*) (Figure 2D). Our *in vitro* data are in line with a stimulatory effect of Ghrelin and a Ghrelin-mimetic towards TRPM6/7 which can be blocked by the GHSR inhibitor (D-Lys^3^)-GHRP6 pointing to a specific role of GHSR in TRPM6/7 stimulation.

### 3.3 Ghrelin enhances TRPM6/7 via Gα_s_ and protein kinase A (PKA)

**FIGURE 3:**
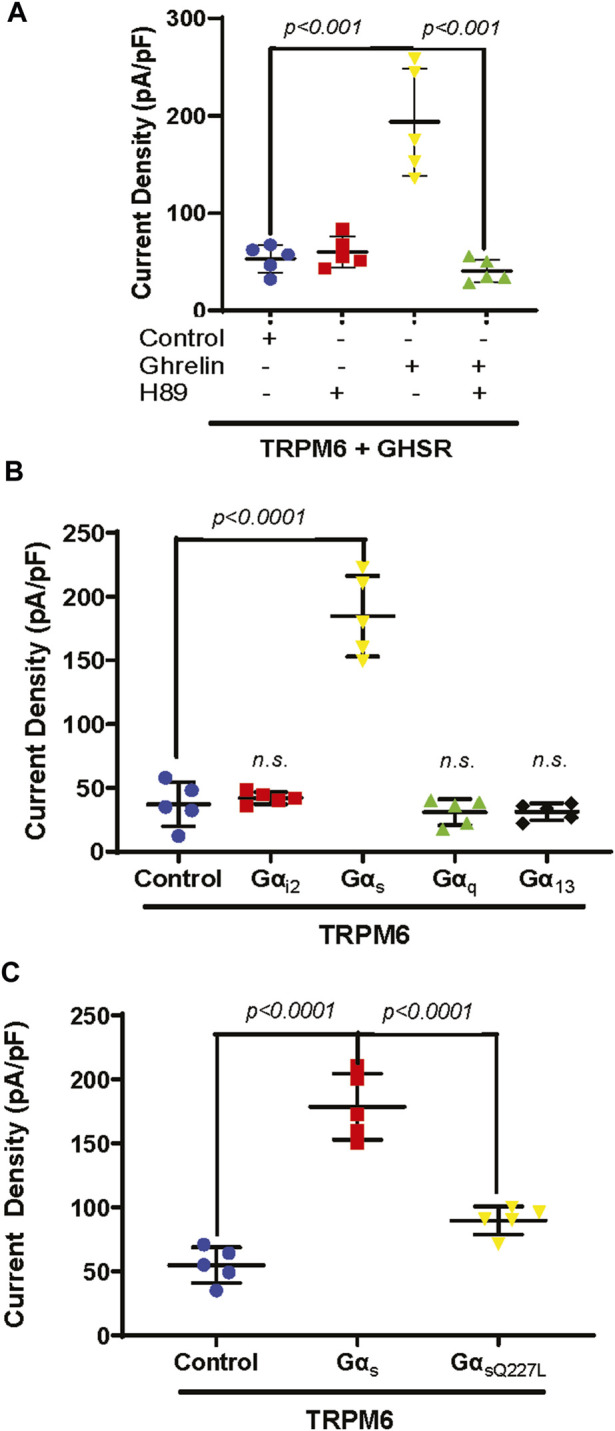
**(A)** The PKA inhibitor H89 inhibits TRPM6/7 stimulation by Ghrelin. H89 has no effect on TRPM6/7 current density by itself (TRPM6+GHSR + control 53.2 ± 14.2 pA/pF vs. TRPM6+GHSR + control + H89 60.3 ± 15.8 pA/pF, *n. s.*). Ghrelin activates TRPM6/7 current density and addition of H89 blocked the Ghrelin-mediated TRPM6 activity (TRPM6+GHSR + Ghrelin 193.5 ± 55.1 pA/pF vs. TRPM6+GHSR + Ghrelin + H89 40.6 ± 11.8 pA/pF, *p < 0.001*). **(B)** Only Gα_s_ enhances TRPM6/7 current density (TRPM6+Gα_s_ 184.7 ± 31.7 pA/pF vs. TRPM6+control 37.2 ± 17.3 pA/pF, *p < 0.0001*) but not Gα_i2_, Gα_q_, or Gα_13_ (TRPM6+Gα_i2_ 42.2 ± 4.7 pA/pF vs. TRPM6+ Gα_q_ 31 ± 10.3 pA/pF vs. TRPM6+ Gα_13_ 31.3 ± 6.7 pA/pF, *n. s.*, n = 5 each group). **(C)**. While Gα_s_ activates TRPM6 current density the Gα_s_ mutant Q227L cannot stimulate TRPM6 current density (TRPM6+control vs. TRPM6+Gα_s_ vs. TRPM6+Gα_sQ227L_ = 54.9 ± 13.9 pA/pF vs. 178.6 ± 25.9 pA/pF vs. 89.7 ± 10.9 pA/pF, *p < 0.0001,* n = 5 each group).

Previous *in vivo* experiments demonstrated that Ghrelin upregulates ENaC in the kidney tubule via protein kinase A (PKA) (Kemp et al., 2013; Kemp et al., 2019). Furthermore, TRPM6 and TRPM7 are also stimulated by cAMP and PKA (Takezawa et al., 2004; Blanchard et al., 2015). Therefore, we tested if PKA mediates Ghrelin stimulation of TRPM6 applying the PKA inhibitor H89. As anticipated Ghrelin enhanced TRPM6/7 whole-cell current density (*p < 0.001*) whereas the addition of H89 alone to TRPM6 and GHSR transfected cells had no significant effect (Figure 3A). When HEK293 cells transfected with TRPM6 and GHSR were treated with Ghrelin and H89 the Ghrelin stimulation of TRPM6/7 was abrogated (*p < 0.001*) (Figure 3A).

**FIGURE 4:**
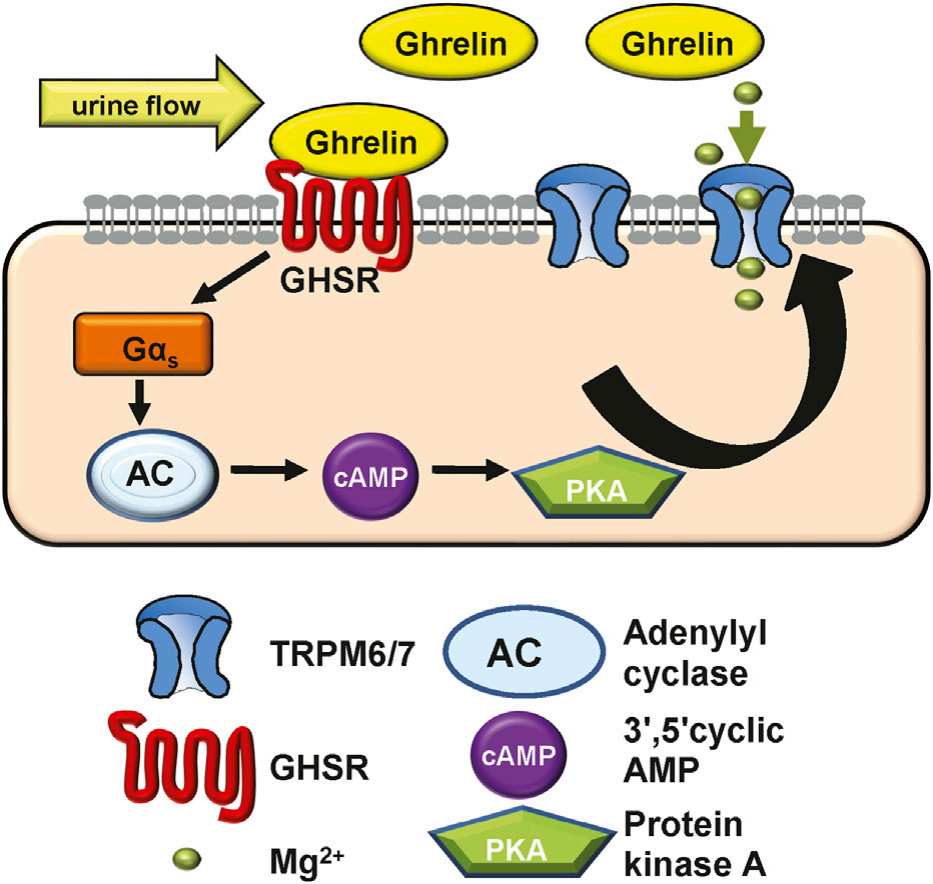
Model of how Ghrelin may stimulate TRPM6/7 based on our *in vitro* data. Given its low molecular weight Ghrelin may be filtered or synthesized in the kidney. Ghrelin binds to the GHSR receptor (which we hypothesize to be apically located) to induce Gα_s_ signalling, activate adenylyl cyclase and raise cAMP levels. These result in PKA activation which then stimulates the Mg^2+^ channel TRPM6/7 and allows for more Mg^2+^ absorption. In Ghrelin deficiency the lack of TRPM6/7 stimulation may contribute to osteopenia. In addition, our *in vivo* experiments show that GHSR enhances transcription and translation of magnesiotropic genes after caloric restriction.

GHSR is a member of the extensive seven transmembrane segment (7TM) receptor family which transduce extracellular signals to elicit intracellular responses via intracellular signaling utilizing G-protein-dependent and -independent mechanisms (Sivertsen et al., 2013). Given the requirement of PKA for Ghrelin stimulation of TRPM6/7 we were interested if G-protein signaling is involved in this signaling pathway. When we transfected plasmids encoding either Gα_i2_, Gα_s_, Gα_q,_ or Gα_13_ together with TRPM6 we only found enhancement of TRPM6/7 whole-cell current density with Gα_s_ (*p < 0.0001*) (Figure 3B). This stimulatory effect was not found when we transfected the Gα_s_ mutant Q227L (Figure 3C). These findings are consistent with Ghrelin-mediated TRPM6/7 stimulation via Gα_s_ and PKA (Figure 4).

### 3.4 Ghrelin and GHSR mRNAs are localized in TAL and DCT

**FIGURE 5:**
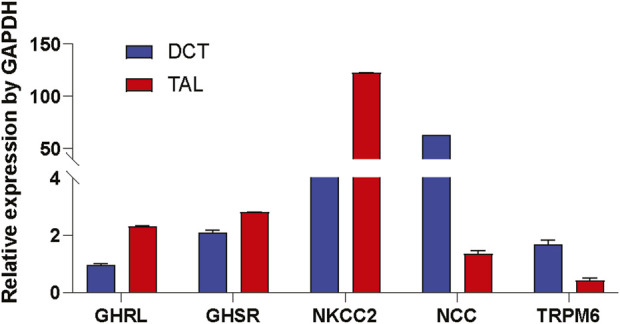
Ghrelin (GHRL) and GHSR mRNA is detected in microdissected TAL and DCT. In microdissected TAL and DCT from WT mice mRNAs of marker genes for the TAL (e.g., NKCC2) and DCT (e.g., NCC and TRPM6) were identified. Expression of mRNA is normalized to GAPDH. GHRL mRNA was two-fold higher in TAL vs. DCT. GHSR mRNA expression was similar in TAL and DCT. As anticipated NKCC2 was most abundant in TAL, whereas NCC and TRPM6 were strongly expressed in the DCT. n = 10 tubules for each group.

After identifying a signaling mechanism for TRPM6/7 stimulation by Ghrelin and GHSR *in vitro*, we tested the physiological significance by studying if the mRNA encoding Ghrelin and GHSR is localized in the same nephron segment as the mRNA for TRPM6. TRPM6 mRNA has been detected in human and rat colon and kidney. In the kidney, TRPM6 mRNA is most abundant in the DCT whereas TRPM7 is ubiquitously expressed (Schlingmann et al., 2002; Walder et al., 2002). In microdissected WT mouse tubule, we found similar levels of GHSR mRNA in TAL and DCT (Figure 5) but Ghrelin mRNA was almost two-fold higher localized in the TAL compared to the DCT. As anticipated NKCC2 and NCC mRNA levels were most abundant in the TAL and DCT, respectively. While TRPM6 mRNA abundance was highest in the DCT in accordance with previous publications there was also 25% of TRPM6 mRNA abundance (compared to DCT) found in the TAL. These results point to a similar localization of GHSR in TAL and DCT. The presence of Ghrelin and GHSR mRNA together with TRPM6 mRNA along the mouse nephron supports a possible physiological role of our identified signaling pathway.

### 3.5 A GHSR reporter mouse shows GHSR expression in TAL but not in the DCT

**FIGURE 6:**
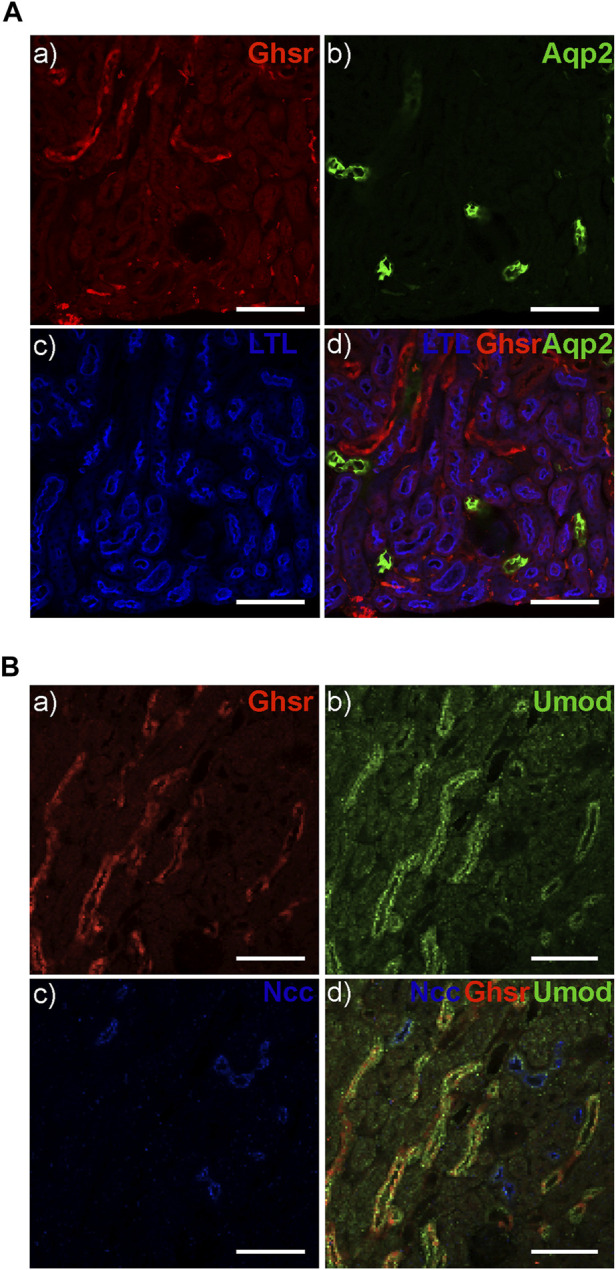
The GHSR-eGFP reporter mouse displays GHSR-eGFP signal in TAL but not in DCT. **(A)** The GHSR-eGFP signal (red) **(A)** does not overlap with the signal for Aqp2 (green) as a marker for the collecting duct **(B, D)** or Lotus Tetragonolobus Lectin (LTL) (blue) as a marker for the proximal tubule **(C, D)**. **(B)** The GHSR-eGFP signal (red) **(A)** overlaps with the signal for Uromodulin (Umod) (green) as a marker for the TAL **(B, D)** but not with the signal for NCC (blues) as the marker for the DCT **(C, D)**. Arrows depict GHSR localization in the Umod-stained TAL **(D)**. Scale bar represents 100 µm.

After identifying Ghrelin, GHSR, and TRPM6 mRNA in the DCT we were interested in the localization of the GHSR protein along the mouse tubule. Given the unavailability of validated, specific anti-GHSR antibodies, we examined the eGFP signal in the reporter GHSR-eGFP mouse model by immunofluorescence. We detected GHSR-eGFP signal within the kidney thus supporting a role of Ghrelin in the kidney. Different tubular markers were used to identify the different tubular segments. Staining for Lotus Tetragonolobus Lectin (LTL) as a marker for the proximal tubule and for Aquaporin 2 (Aqp2) as a marker for the collecting duct did not reveal colocalization with the GHSR-eGFP signal (Figure 6A). Colocalization of the GHSR-eGFP signal was only found with Uromodulin, suggesting GHSR-eGFP localization in the TAL but not with NCC in the DCT (Figure 6B). These findings point to GHSR localization in the mouse TAL but not in the DCT. The reason for the discrepancy with mRNA signal could be due to the nature of the reporter alleles, which do not always accurately represent endogenous expression patterns.

### 3.6 GHSR-null mice develop hypermagnesuria at 9 months of age

**FIGURE 7:**
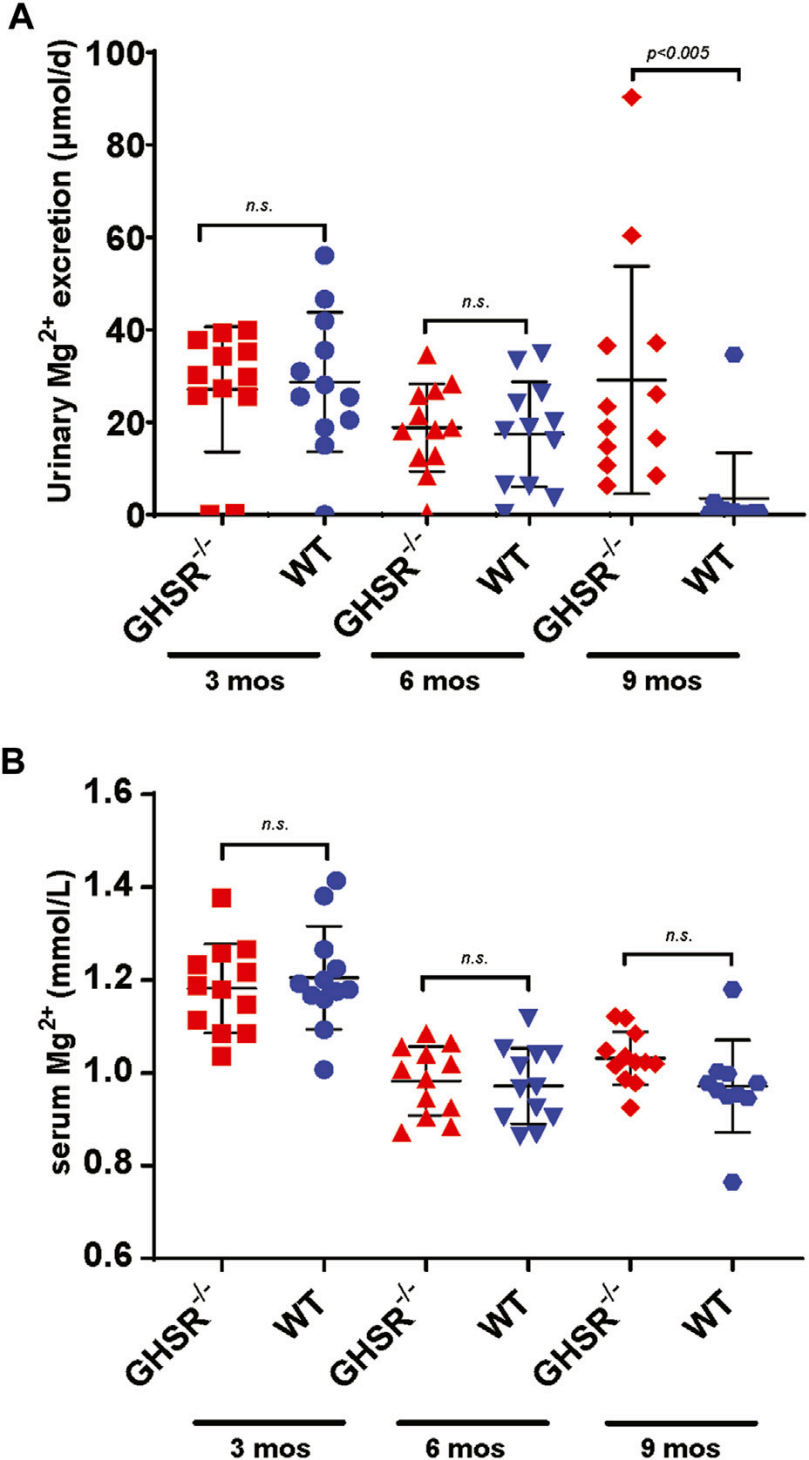
At baseline, GHSR-null mice develop hypermagnesuria at 9 months of age. **(A)** At 3 and 6 months of age WT and GHSR-null mice do not show any significant differences in urinary Mg^2+^ excretion (3 months urinary Mg^2+^ excretion GHSR^−/−^ 27.1 ± 13.6 vs. WT 28.7 ± 15.1 μmol/d, *n. s.*; 6 months urinary Mg^2+^ excretion GHSR^−/−^ 18.8 ± 9.5 vs. WT 17.4 ± 11.4 μmol/d, *n. s.*). At 9 months of age the GHSR-null mice showed a significantly higher urinary Mg^2+^ excretion compared to WT mice (9 months urinary Mg^2+^ excretion GHSR^−/−^ 29.1 ± 24.6 vs. WT 3.5 ± 9.8 μmol/d, *p < 0.005*; n = 12 for each group). **(B)** Serum Mg^2+^ levels were not significantly different in 3-, 6-, and 9-month-old GHSR-null vs. WT mice (3 months serum Mg^2+^ GHSR^−/−^ 1.18 ± 0.1 vs. WT 1.21 ± 0.1 mmol/L, *n. s.*; 6 months serum Mg^2+^ GHSR^−/−^ 0.98 ± 0.07 vs. WT 0.97 ± 0.08 mmol/L, *n. s.*; 9 months serum Mg^2+^ GHSR^−/−^ 1.03 ± 0.06 vs. WT 0.97 ± 0.1 mmol/L, *n. s.*; n = 10–12 for each group).

To better comprehend the role of Ghrelin in tubular Mg^2+^ homeostasis we analyzed GHSR-null mice at baseline at 3, 6, and 9 months of age regarding serum and urine Mg^2+^ excretion. Daily urinary Mg^2+^ excretion was not significantly different at 3 and 6 months (Figure 7A). Only at 9 months GHSR-null mice displayed a significantly higher urinary Mg^2+^ excretion (Figure 7A). Two-way ANOVA was calculated for the urinary Mg^2+^ excretion from 3 to 9 months using GraphPad Prism nine and resulted in a *p*-value of 0.0002. Food intake did not show any significant difference at 3, 6, and 9 months (Supplementary Figure S2). Serum Mg^2+^ levels were not significantly different in WT and GHSR-null mice at 3, 6, and 9 months (Figure 7B). For the serum Mg^2+^ from 3 to 9 months the two-way ANOVA resulted in a *p*-value of 0.0001. These data do not point to a significant role of Ghrelin in acute tubular Mg^2+^ regulation at baseline but to a possible age effect on renal Mg^2+^ homeostasis.

### 3.7 Calorically restricted GHSR-null mice develop lower serum Mg^2+^ levels and hypermagnesuria

**FIGURE 8:**
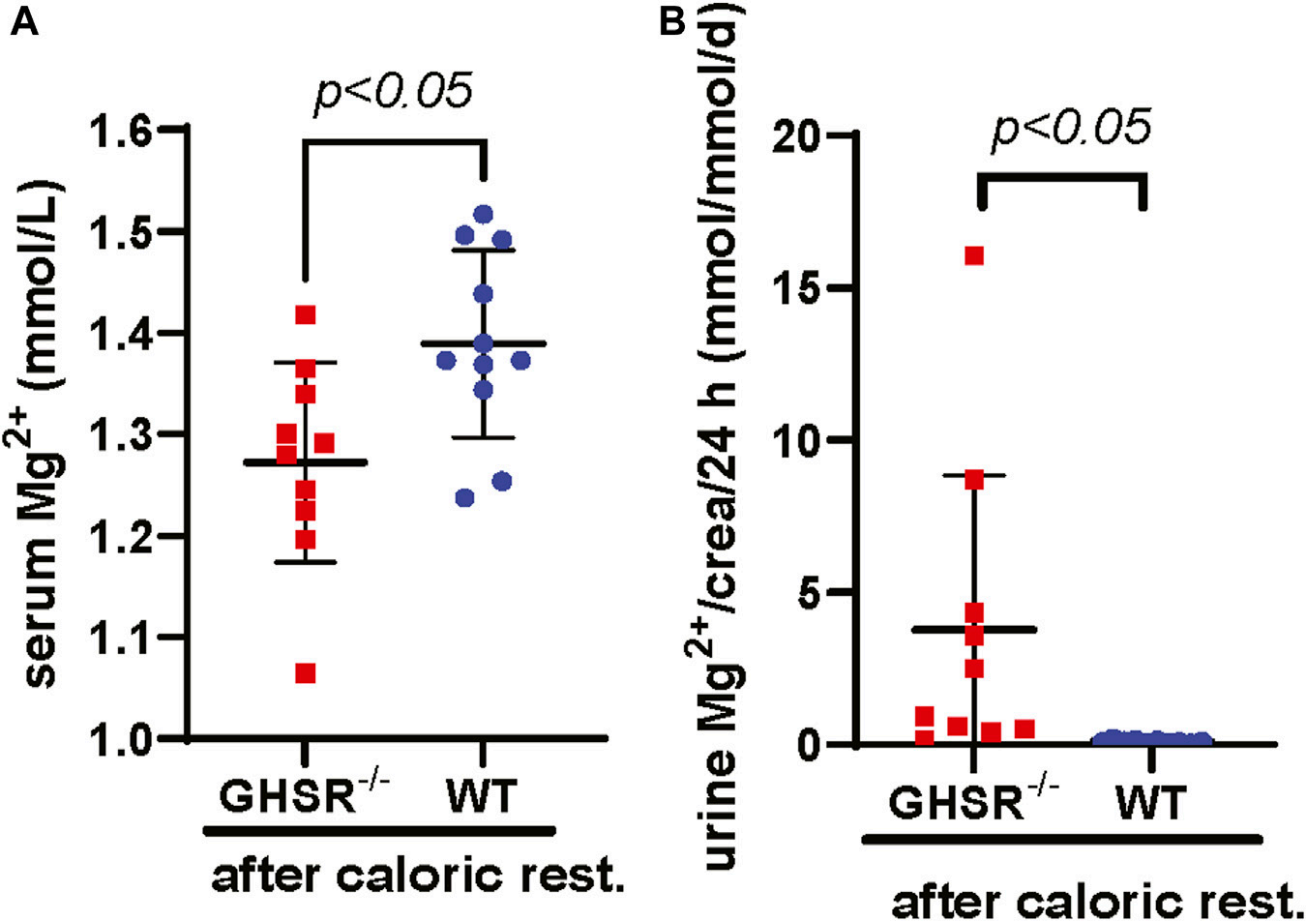
Calorically restricted GHSR-null mice develop lower serum Mg^2+^ and hypermagnesuria. **(A)** After caloric restriction for a total of 9 days in 3-month-old GHSR-null and WT mice we observe significantly higher serum Mg^2+^ levels in WT animals (serum Mg^2+^ GHSR^−/−^ 1.27 ± 0.11 vs. WT 1.39 ± 0.09 mmol/L, *p < 0.05,* n = 10–11 for each group). **(B)** After caloric restriction, we find significantly higher daily urinary Mg^2+^ excretion corrected for urinary creatinine in GHSR-null mice (urinary Mg^2+^ excretion/crea/d GHSR^−/−^ 4.3 ± 6.1 vs. WT 0.1 ± 0.1 mmol/mmol/d, *p < 0.05,* n = 10–11 for each group). Caloric rest., caloric restriction; crea, creatinine.

Because of the physiological role of Ghrelin as a mediator of apatite and higher Ghrelin levels during hunger we asked ourselves if the Ghrelin effect on tubular Mg^2+^ regulation would manifest when the GHSR-null mice undergo caloric restriction. After assessing caloric intake for 5 days, food intake was reduced to 40% over 8 days followed by a day of complete fasting (Zhang et al., 2015). This caloric restriction protocol led GHSR-null mice to develop significantly lower serum Mg^2+^ (*p < 0.05*) and higher urinary Mg^2+^ (*p < 0.05*) excretion while WT mice displayed complete suppression of urinary Mg^2+^ excretion (Figure 8A&B). This result is consistent with a role of Ghrelin in tubular Mg^2+^ homeostasis during extreme situations such as caloric restriction.

### 3.8 Calorically restricted WT mice upregulate renal magnesiotropic genes compared to GHSR-null mice

**FIGURE 9:**
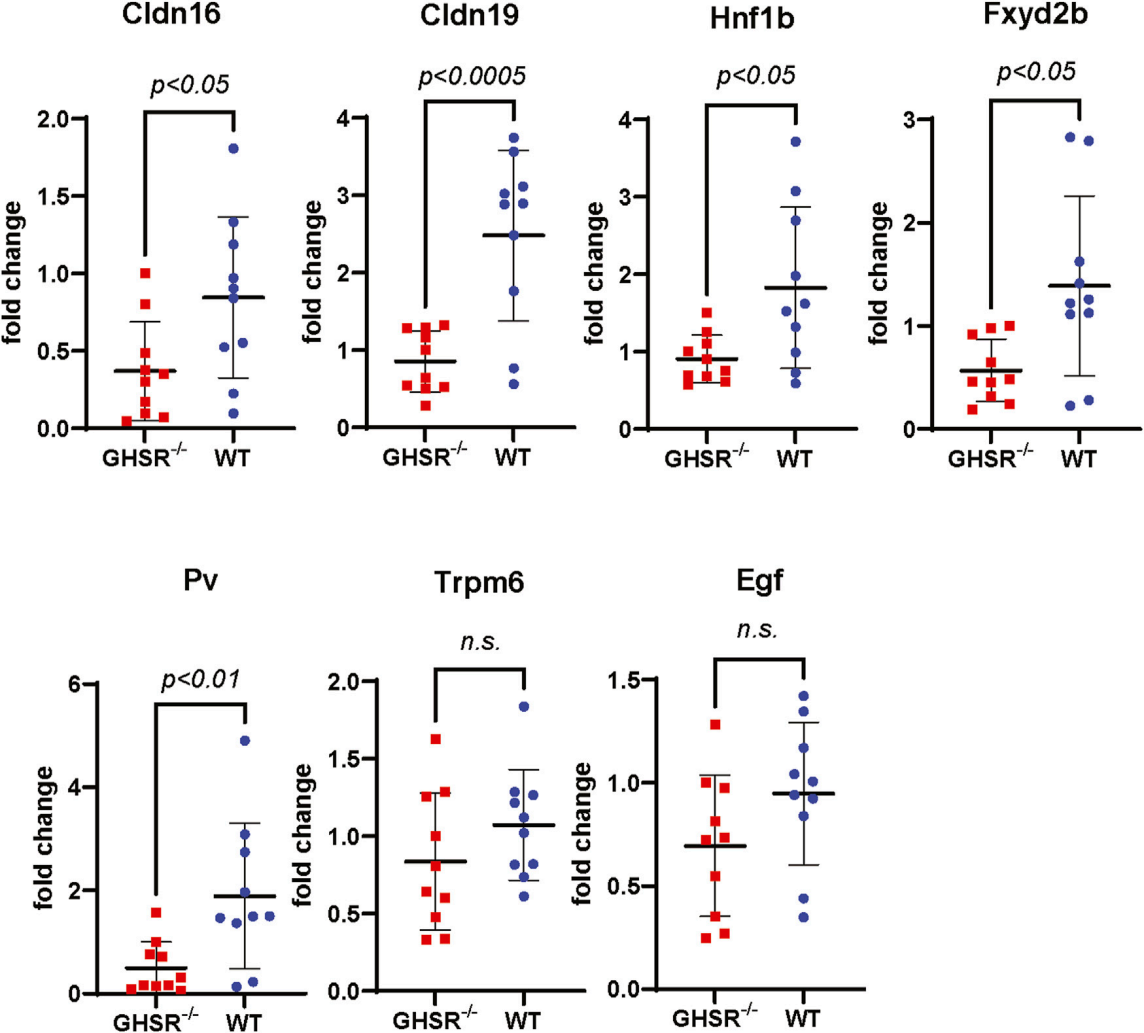
Transcription of magnesiotropic genes in GHSR-null and in WT mice after caloric restriction. Expression of mRNA levels of magnesiotropic genes such as Claudin-16 (Cldn16), Claudin-19 (Cldn19), Hnf1b, Fxyd2b, and Parvalbumin were significantly lower in GHSR-null mice compared to WT mice. No significant differences were found for Trpm6 or epidermal growth factor mRNA levels (Egf) after caloric restriction.

To better understand how caloric restriction and lack of Ghrelin influences tubular Mg^2+^ regulation we performed qPCR of kidney tissue from WT and GHSR-null mice to test if long-term caloric restriction may influence transcription of magnesiotropic genes. Our qPCR data show a significantly lower upregulation of the magnesiotropic genes Claudin-16 (Cldn16), Claudin-19 (Cldn19), HNF1B (Hnf1b), FXYD2B (Fxyd2b), and Parvalbumin (Pv), but no significant difference in mRNA expression was detected for TRPM6 (Trpm6) or EGF (Egf) (Figure 9). Immunoblotting of Trpm6 and Claudin-16 protein revealed significantly higher protein abundance in kidney lysate of WT mice compared to GHSR-null mice (Supplementary Figure S3). Our data point to a possible role of Ghrelin and GHSR in regulating transcription and translation of magnesiotropic genes during prolonged caloric restriction. Given the lack of Cldn16 and Cldn19 mRNA upregulation in GHSR-null mice after caloric restriction (Figure 9) the TAL may also play an important role in Ghrelin-mediated regulation of renal Mg^2+^ homeostasis.

## 4 Discussion

Bone disease has been described in post-gastrectomy patients for over 30 years (Klein et al., 1987; Bisballe et al., 1991). Osteopenia after bariatric surgery involves mechanical and nutritional components (Folli et al., 2012). Obesity, in itself, is a protective factor against osteoporosis, and once weight loss occurs, bone density decreases due to mechanical unloading (Khosla et al., 1996; Barrera et al., 2004). Another component is impaired calcium and fat-soluble vitamin absorption in the duodenum and jejunum–dependent on whether they are bypassed by the procedure–which may aggravate bone loss (Khosla et al., 1996; Barrera et al., 2004; De Prisco and Levine, 2005). However, most of the clinical studies were small in size and larger studies were not able to confirm significant differences in calcium, PTH, or vitamin D levels in patients after bariatric surgery (Bano et al., 1999; Vilarrasa et al., 2009). Interestingly, vitamin D supplementation in rats after gastric bypass surgery was not sufficient to ameliorate decreased bone volume (Stemmer et al., 2013). Another component in some patients is the development of hyperparathyroidism (Hamoui et al., 2003). Even though secondary hyperparathyroidism due to low vitamin D levels and mechanical unloading from weight loss are the most commonly quoted mechanisms for osteopenia after bariatric surgery, data to support these hypotheses are scant (Yu, 2014). New hypotheses involving crosstalk between the bone and gastrointestinal hormonal systems have evolved recently (Folli et al., 2012; Quercia et al., 2014; Malkani, 2015). The gastric hormone Ghrelin is known to increase bone mass when given to rats (Fukushima et al., 2005; Choi et al., 2013). In *ex vivo* cultures, Ghrelin inhibits osteoclastogenesis, and prevents apoptosis in osteoblastic cells, and GHSR-null mice have reduced bone volume (Maccarinelli et al., 2005; van der Velde et al., 2012; Liang et al., 2013). While weight loss, possible malabsorption, and Ghrelin are well-studied regarding their effects on bone metabolism, Mg^2+^ is a frequently overlooked ion in bone metabolism but seems equally important (Boskey et al., 1992; Rude et al., 2003; Rude et al., 2004; Rude and Gruber, 2004).

While we did not identify any stimulation of TRPV5 channels by Ghrelin (Figure 1A), we demonstrated a dose-dependent effect of Ghrelin regarding the Mg^2+^ channel TRPM6/7 (Figure 2A) which is specific to TRPM6/7 and not TRPV5. We found upregulation of TRPM6/7 by Ghrelin dosages as low as 1 nM (Figure 2A). Stimulation of TRPM6/7 by Ghrelin was blocked by the GHSR inhibitor (D-Lys^3^)-GHRP6 (Figure 2B) and the Ghrelin-mimetic D-Trp^7^, Ala^8^, D-Phe^10^)-α-MSH (6–11) amide also resulted in TRPM6/7 stimulation (Figure 2D) thus supporting a role for Ghrelin and GHSR effect on TRPM6/7. In humans, serum Ghrelin levels range approximately between 100 and 500 pmol/L, dependent on the fasting status (Janssen et al., 2001; De Souza et al., 2004). Given the fact that Ghrelin is almost twenty-fold elevated in kidney tissue and three-fold higher in human urine compared to serum levels, this would be consistent with upregulation of TRPM6/7 by Ghrelin in a physiologically relevant concentration range (Mori et al., 2000; Aydin et al., 2008). Our *in vitro* data point to Ghrelin - either filtered in the glomerulus (given its low molecular weight) or synthesized in the kidney (given our mRNA data) (Figures 4, 5) - stimulating GHSR which signals through Gα_s_ (Figure 3B), adenylyl cyclase, and cAMP to activate PKA (Figure 3A) and stimulate TRPM6/7 (Figure 4). This pathway is in line with previous publications describing Ghrelin to enhance the Na^+^ channel ENaC via PKA and TRPM6 is also stimulated by PKA (Blanchard et al., 2015; Kemp et al., 2019). It seems likely, that Ghrelin could also enhance TRPM6/7 in the gastrointestinal tract via PKA. We cannot exclude additional or alternative downstream signaling involving GHSR and TRPM6/7 activation, as well as possible activation of endogenous TRPM7 by PKA which has been previously published (Takezawa et al., 2004). Another signaling pathway was shown for the orexigenic effect of Ghrelin: activation of GHSR in neurons of the arcuate nucleus of the hypothalamus involves activation of sirtuin-1 (Sirt1), which deacetylates p53 and forkhead box protein O1 (FoxO1), thus making both transcription factors available to regulation of gene transcription (Howard et al., 1996; Kojima et al., 1999; Velasquez et al., 2011). Subsequently, AMP-activated protein kinase (AMPK) is phosphorylated which modifies mRNA expression of several metabolic enzymes and mitochondrial respiration (Velasquez et al., 2011). Our data also point to a transcriptional and translational role of GHSR towards magnesiotropic genes during caloric restriction *in vivo* (Figure 9). We cannot comment on whether GHSR stimulates TRPM6/7 conductance, open probability, or cell surface abundance.

We focused on the effect of acylated Ghrelin (AG) which represents about 10% of the total circulating Ghrelin (Ariyasu et al., 2001). Ghrelin is synthesized as pre-pro-Ghrelin and pro-Ghrelin is cleaved to form unacylated (e.g., des-acyl Ghrelin abbreviated as DAG) and AG which binds to GHSR. The enzyme Ghrelin-O-acyltransferase (GOAT) converts DAG to AG (Yang et al., 2008). While DAG is the dominant form of Ghrelin in plasma little is known about its function. Until recently, DAG was considered to be inactive as it does not bind to GSHR. But recent data point to DAG participating in multiple processes, possibly as an independent hormone sometimes even counteracting AG (Broglio et al., 2004; Pacifico et al., 2009; Benso et al., 2012; Delhanty et al., 2014; Shimada et al., 2014). Our studies only focus on AG mediated actions via GHSR and cannot comment on any involvement of DAG in tubular Mg^2+^ absorption.

Our microdissection data support similar localization of GHSR mRNA in both the TAL and DCT, whereas Ghrelin mRNA is two-fold higher in TAL vs. DCT (Figure 5). The understanding of the physiological roles of Ghrelin and GHSR has been complicated by notoriously unspecific antibodies. This problem has been in part addressed by reporter mice. Our immunofluorescent studies using the GHSR-eGFP mice did not detect any GHSR signal in the DCT. However, applying such model systems (e.g., GHSR-eGFP), a role for GHSR in the kidney has been previously demonstrated by immunofluorescent and immunohistochemistry studies in distal tubules (Venables et al., 2011). The authors used the same GHSR reporter mouse as in our experiments but on a different background. Despite the different genetic background of our GHSR-eGFP reporter mouse model, our results confirmed a role for GHSR in the TAL but not in the proximal tubule, the DCT, or the collecting duct as also outlined in the previous publication (Venables et al., 2011). Venables et al. showed a fluorescent signal in the outer medulla and the straight parts of distal tubules in the medulla. This localization data was confirmed by *in situ* hybridization histochemistry. No GHSR signal was found in the glomerulus, the proximal tubule, or the collecting duct (Venables et al., 2011). Colocalization of GHSR was only shown with UMOD with apical staining for UMOD and more cytoplasmic localization of GHSR. Unfortunately, the study lacked any other specific tubular marker than UMOD. Therefore, similar to our findings, the authors showed GHSR localization in the cytoplasm of the TAL but no proof of GHSR localization in the DCT. Retrospectively, the authors’ use of the term “distal tubule” appears unfortunate because it is so easily confused with the DCT. Finally, given the results from the caloric restriction in mice (Figure 8) it is possible that caloric restriction could enhance GHSR protein expression. We did not study the effect of caloric restriction on renal GHSR-eGFP expression.

While we and others could not detect any GHSR localization in the collecting duct (Figure 6A) there still seems to be a role for GHSR regarding ENaC stimulation in the collecting duct resulting in obesity- and angiotensin-II-mediated hypertension (Kemp et al., 2013; Kemp et al., 2014; Kemp et al., 2018). Using GHSR antibodies in rat kidney a GHSR signal colocalized with AQP2. Weaknesses of this study were that GHSR antibodies were used which are frequently unspecific and that Ghrelin was provided at 0.3 and 3 μg/min (Kemp et al., 2013). This may have likely resulted in supraphysiological dosages given that human serum Ghrelin levels range between 100 and 500 pmol/L raising concerns regarding the physiological relevance (Janssen et al., 2001; De Souza et al., 2004; Kemp et al., 2013). However, multiple lines of additional studies (for example, in uni-nephrectomized mice fed with a high-fat diet applying the GHSR antagonist (D-Lys^3^)-GHRP6) support a likely role for Ghrelin in tubular Na^+^ regulation and blood pressure control in the collecting duct by mediating ENaC translocation signaling through PKA and activating microtubules (Kemp et al., 2014; Kemp et al., 2018; Kemp et al., 2019). There seems to be a discrepancy between using the GHSR-eGFP reporter mouse model and GHSR antibodies, but the additional lines of functional studies targeting Ghrelin and GHSR raise questions how accurate the GHSR-eGFP mouse is or if we may have missed more subtle GHSR localization in the DCT or the collecting duct.

While we did not detect any major abnormalities in daily urinary Mg^2+^ excretion or serum Mg^2+^ at three or 6 months, there was a significantly higher daily urinary Mg^2+^ excretion at 9 months of age for the GHSR-null mice compared to the WT animals (Figure 7). However, this was not accompanied by a lower serum Mg^2+^ level. It remains unclear how relevant the higher urinary Mg^2+^ excretion at 9 months is in the context of a normal serum Mg^2+^ level. This finding may point to a role for Ghrelin in tubular Mg^2+^ absorption in aging animals but not in younger animals at baseline. Lower serum Mg^2+^ levels and higher urinary Mg^2+^ have been described in older human individuals (Gullestad et al., 1994; Barbagallo et al., 2009; Gautam and Khapunj, 2021). In our WT mice the serum Mg^2+^ concentration was not significantly different at 3 months and there was no significant decrease of serum Mg^2+^ levels between 6 and 9 months (Figure 7). This was associated with a higher daily urinary Mg^2+^ excretion at the age of 3 months, but daily urinary Mg^2+^ concentration dropped with increasing age. However, the extreme challenge of caloric restriction resulted in the GHSR-null mice in a significantly lower serum Mg^2+^ and a higher daily urinary Mg^2+^ excretion (Figure 8). As an explanation we identified a lower gene transcription and protein expression of magnesiotropic genes in GHSR-null mice (Figure 9; Supplementary Figure S3). Another interpretation of this finding could be that GHSR-null mice also display an increase in tubular damage and renal ROS levels, renal dysfunction, and changes in renal senescence and fibrosis which could also contribute to higher urinary Mg^2+^ losses (Fujimura et al., 2014). But we would expect tubular damage to also affect baseline serum Mg^2+^ and baseline urinary Mg^2+^ excretion (Figure 7) which was not our finding. Because our data only included male mice, we cannot comment on the GHSR effect in female mice.

Mainstream understanding is that the TRPM6 channel is mostly localized in the DCT with weaker signals in the proximal tubule and collecting duct based on TRPM6 cDNA in microdissected rat nephrons whereas TRPM7 is ubiquitously expressed (Runnels et al., 2002; Schlingmann et al., 2002). These findings were supported in human and mouse kidney examining mRNA for TRPM6 (Walder et al., 2002). In contrast, TRPM6 protein was only detected in DCT of WT mice (Voets et al., 2004). One possibility of these discrepancies can be impurities of microdissection and subsequent qPCR. As anticipated, our microdissection studies showed strong abundance of NKCC2 mRNA in TAL, whereas NCC and TRPM6 were the highest abundant in DCT. However, the 25% mRNA abundance of TRPM6 in the TAL (compared to DCT) was surprising to us (Figure 5). These 25% of TRPM6 in the TAL may not be relevant at baseline (Figure 7) but could perhaps become significant under extreme challenges such as caloric restriction (Figure 8). Furthermore, we cannot exclude an effect of caloric restriction on TRPM7 mRNA gene expression. Given our mRNA and protein expression data from the caloric restriction (Figure 9) it also appears plausible that the TAL may play a role in Ghrelin-mediated renal Mg^2+^ regulation. Alternatively, one may also consider a possible impurity of the microdissection (Figure 5). However, as known for Na^+^ and Ca^2+^ transport there are smaller differences between rodents such as mice and rat compared to humans (Loffing and Kaissling, 2003). Considering that we detected the strongest immunofluorescent GHSR signal in the TAL our qPCR data under caloric restriction make sense with the strongest effect on TAL genes such as Claudin-16 and Claudin-19 (Figure 9).

Overall, we show that Ghrelin stimulates TRPM6/7 via GHSR and Gα_s_-PKA signaling *in vitro*. However, the physiological significance of this mechanism is unclear given the immunofluorescent GHSR signal in the TAL while Ghrelin and GHSR mRNA is localized in the TAL and the DCT. We hypothesize that enhanced Ghrelin secretion during hunger periods may help the body to maintain Mg^2+^ homeostasis by increasing renal tubular Mg^2+^ absorption via GHSR. Calorically restricted GHSR-null mice had significantly elevated urinary magnesium excretion and lower serum magnesium levels compared to WT mice. This may be mediated by Ghrelin-upregulation of TRPM6 in the TAL and/or upregulation of magnesiotropic genes. In the future, bariatric surgery, and side effects such as osteopenia may become less common given the evolution of GLP-1 agonists as new therapy for weight loss.

## Data Availability

The original contributions presented in the study are included in the article/Supplementary Material, further inquiries can be directed to the corresponding author.
