# Supplementary figures and images for: Ghrelin enhances tubular magnesium absorption in the kidney
Source: Front Physiol. 2024 Apr 4;15:1363708. doi: 10.3389/fphys.2024.1363708 (PMC11024433; doi:10.3389/fphys.2024.1363708)

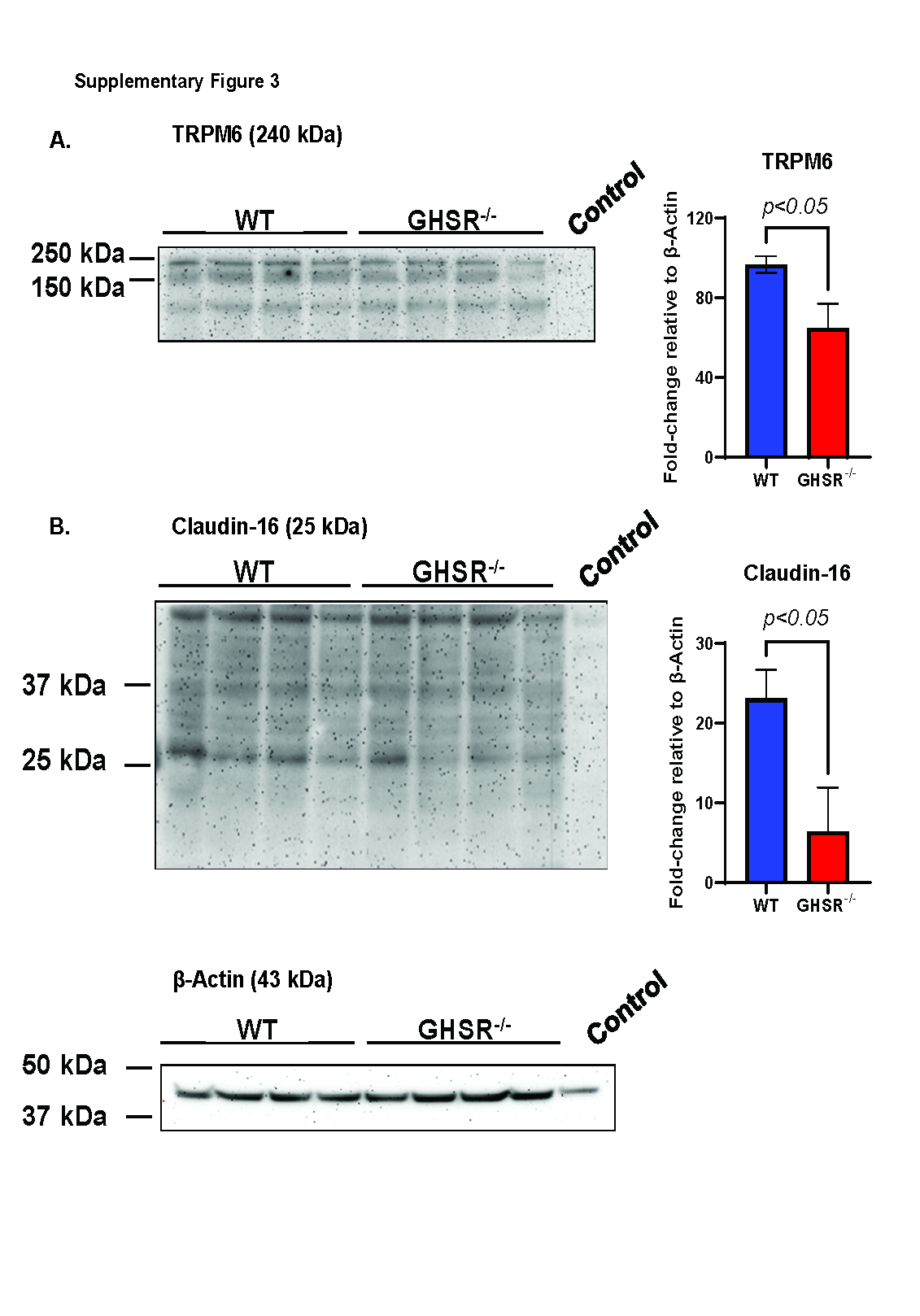

Supplement: Supplementary file 1 [file Image3.TIFF]

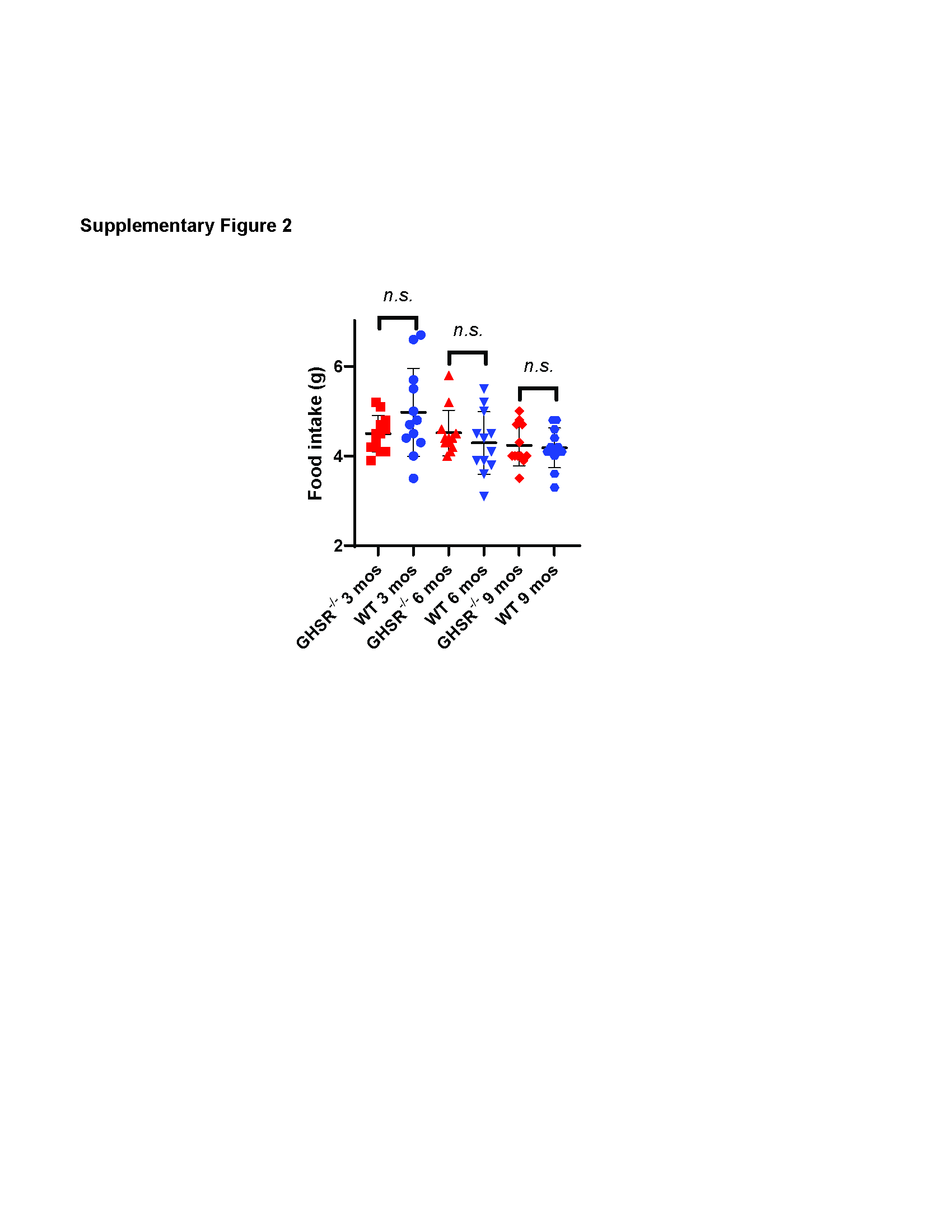

Supplement: Supplementary file 2 [file Image2.TIF]

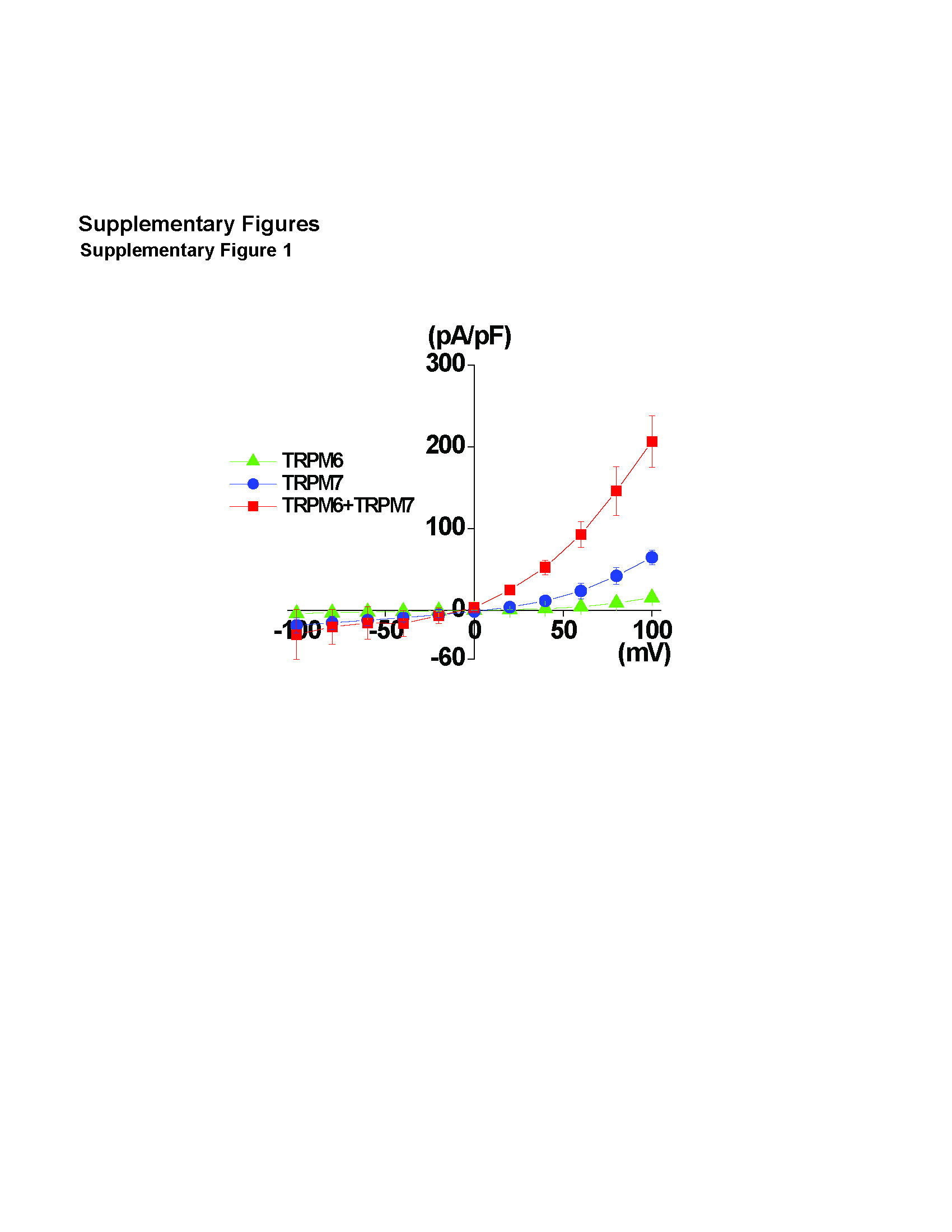

Supplement: Supplementary file 3 [file Image1.TIF]
